# A Case Report of Psychogenic Non-epileptic Seizures in a 29-Year-Old Male With Schizophrenia

**DOI:** 10.7759/cureus.37078

**Published:** 2023-04-03

**Authors:** Evan M Banks, Maggie M Plattes

**Affiliations:** 1 Medical School, University of Minnesota, Minneapolis, USA; 2 Psychiatry, Hennepin Healthcare, Minneapolis, USA

**Keywords:** psychogenic non-epilipetic seizures, young adult male, suicide risk, neurology and epilepsy, schizophrenia and other psychotic disorders

## Abstract

Psychogenic non-epileptic seizures (PNES) involve episodes of movement, sensation, or behaviors that may appear clinically similar to epileptic seizures but without cortical electroencephalographic activity that defines epileptic seizures. This case report involves a 29-year-old male with a history of type I diabetes mellitus, schizophrenia, and a prior suicide attempt via insulin overdose. He was admitted to the emergency department after being found unresponsive on the floor in his bedroom. Given the nature of his prior suicide attempt, he was initially treated for hypoglycemic coma. After arrival at the emergency department, he was noted to have normal blood glucose but displayed symptoms of acute psychosis and was transferred to the behavioral health unit, where subsequent paroxysmal episodes with seizure-like features were observed. He then underwent video-electroencephalography monitoring to evaluate for epilepsy. After no epileptic activity was recorded, he was transferred back to the behavioral health unit and treated for underlying schizophrenia and suspected PNES. After showing gradual improvement on antipsychotic medication, no further seizure-like activity was observed. His stay was complicated by a SARS-CoV-2 infection, which he recovered from without complication, and he was released on day 11. Extensive education was provided for the patient and his family on recognizing the symptoms of PNES and the importance of adherence to antipsychotic medication to avoid psychiatric decompensation and PNES recurrence. This case report highlights the challenge of diagnosing and treating a patient with PNES with underlying psychiatric comorbidities and a history of insulin overdose.

## Introduction

Psychogenic non-epileptic seizures (PNES) present a unique challenge for clinicians to diagnose and treat. They are episodes of movement, sensation, or behaviors that present similar to epileptic seizures but are a type of conversion disorder, or functional neurological disorder, in which psychological distress manifests as physical symptoms [1,2]. Some of these psychological risk factors for PNES include a history of physical or sexual abuse, developmental stressors, post-traumatic stress disorder, dissociative disorders, borderline personality, and neuroticism [3].

Diagnosis of PNES involves a combination of recognizing clinical features and neurological monitoring. Inpatient video-electroencephalography (vEEG) monitoring is the preferred test for diagnosing PNES, especially when differentiating PNES from epileptic seizures [4]. However, a previous study noted a PNES-epilepsy coexistence ratio of 53.69% among all patients with PNES [5]. Therefore, it is important to use clinical features to distinguish between PNES and epileptic seizures. Ali et al. propose a diagnostic approach to PNES that does not rely solely on EEG monitoring [6]. It is essential for providers to take into account the patient’s history of trauma and psychiatric illness and, if witnessed, the characteristics of the seizure episode [6].

Treatment of PNES can be challenging. While antiepileptic medication is not effective for treating PNES specifically, it may still be necessary for treating coexisting epilepsy [7]. Many treatments are focused on addressing the underlying psychological problem. Cognitive behavioral therapy and group therapy are considered first-line treatments [8]. These interventions are efficacious in decreasing seizure-like episode frequency and improving quality of life [8].

Several studies have described the challenge of distinguishing PNES from an epileptic seizure [9-11]. There has also been a case report highlighting the challenge of diagnosing PNES with comorbid schizoaffective disorder [12]. However, no known studies to date have described PNES in a patient with schizophrenia and a history of type I diabetes mellitus with a previous suicide attempt by insulin overdose. The purpose of this case report is to detail patient history gathered on evaluation, clinical phenomena, and neurologic testing that were utilized to distinguish the diagnosis of PNES from a hypoglycemic coma, epilepsy, and catatonic schizophrenia.

## Case presentation

A 29-year-old male with schizophrenia, type I diabetes mellitus, and previous psychiatric admissions for a suicide attempt via insulin overdose was admitted to the emergency department after he was found unresponsive on the floor of his bedroom by his mother. Given his history of insulin overdose, he was initially treated for hypoglycemia with honey and juice. The patient was awake and alert on emergency medical services arrival but with minimal verbal responses. He was able to provide staff with a name and date of birth. Shortly after arrival at the emergency room, the patient began rambling rapidly and nonsensically. He was then restrained after attempting to escape his hospital bed and was given a dose of intravenous droperidol as well as midazolam, with improvement in his acute agitation.

The patient’s past medical history was significant for schizophrenia, diagnosed at age 23, major depressive disorder, diagnosed at age 14, a prior suicide attempt at age 23 via insulin overdose, and previous hospitalization for suicidal ideation. Medication history included sertraline for major depression. Despite his history of schizophrenia, he had never taken an antipsychotic medication. On admission, the patient lived at home with his parents. His parents noted that at baseline, he struggled with paranoia and was unable to maintain employment. The week before admission, they noticed possible signs of psychiatric decompensation, noting that he struggled with insomnia, was making odd, paranoid statements, and spending an increasing amount of time alone in his room. They also reported that he wrote a note to his brother expressing suicidal ideation a few weeks before the current hospitalization.

On examination, the patient’s vital signs were within normal limits, and his physical examination was unremarkable. Other than a blood glucose of 222 mg/dL, all other notable lab values were within normal limits. The patient was deemed medically stable and transferred to a psychiatric unit. On psychiatric evaluation, the patient was oriented to person, time, place, and situation. He was groomed appropriately with a thin body habitus. He appeared restless but answered all questions appropriately with brief responses. His language was intact and clear with normal rate and tone but monotonous prosody and delayed spontaneity. His thought process was intact, clear, and logical, with no expressed suicidal or homicidal ideation. He did endorse auditory hallucinations that were quite disturbing. His affect was flat and subdued. He had impaired recollection of events preceding the current hospitalization. There were no obvious motor abnormalities observed during the psychiatric evaluation.

While in the psychiatric unit, the patient had three witnessed seizure-like episodes on days two, three, and four. Neurology was consulted. A ceribell headband recording during target events was unremarkable. Computed tomography of the head was normal, apart from a known pineal cyst. Computed tomography angiography of the head revealed a possible saccular aneurysm of the supraclinoid segment of the left internal carotid artery, which was deemed not clinically relevant by neurosurgery. Magnetic resonance imaging of the brain showed a stable pineal gland cyst but no epileptic focus. A neurology consult deemed these imaging findings as non-contributory to his current symptoms. With the absence of any known organic cause for his seizure-like episodes, PNES was diagnosed.

The patient was amenable to psychiatric medical intervention for the treatment of schizophrenia and was started on aripiprazole 5 mg daily. In the first few days, he had a low appetite and was not sleeping well. Olanzapine 10 mg once daily was added in place of aripiprazole on day two. On day four, he continued to demonstrate poor sleep and appetite and had another witnessed non-epileptic seizure. During this episode, waxy flexibility and other signs of catatonia were not elicited. Olanzapine was subsequently increased to 10 mg twice daily. He was also given melatonin 3 mg to help with sleep. Once stabilized on this regimen, the patient became more sociable with improved appetite and sleep. After day four, he no longer endorsed distressing auditory hallucinations and did not suffer seizure-like activity. While in the psychiatric unit, the patient contracted SARS-CoV-2 but remained asymptomatic and did not suffer any recurrent PNES episodes before he was discharged home on day 11.

## Discussion

Although PNES may display similarly to epileptic seizures, the exact pathology remains unclear. Many studies have found an association between PNES and psychological trauma, particularly sexual abuse, personality disorder, affective disorder, or a history of post-traumatic stress disorder [3]. Auditory hallucinations in the setting of schizophrenia have been suggested as a possible trigger for PNES [13]. With a PNES episode occurring at the time of distressing auditory hallucinations, one proposed hypothesis regarding the etiology of PNES is that non-epileptic seizures are an example of an exaggerated startle response [13]. In this patient with many psychological risk factors, it is possible that psychiatric decompensation, particularly untreated schizophrenia, played a role in inducing this patient’s first PNES episode. This theory is supported by the fact that this patient did not experience another witnessed PNES episode after being stabilized on antipsychotic medication. Therefore, it is plausible that addressing the underlying auditory hallucinations and other distressing psychological phenomena in this patient may reduce the frequency of recurrent PNES episodes.

An additional consideration in this patient is that schizophrenia often presents with catatonia, making it challenging to differentiate between a catatonic state and a PNES episode [14]. One physical examination maneuver performed on this patient assessed for waxy flexibility, a common sign in catatonia [15]. While performing a physical examination during a witnessed PNES episode, this patient did not demonstrate rigidity in reposturing of his arm. Although the absence of waxy flexibility did not definitely rule out catatonia, it did make catatonic disorder a less likely diagnosis.

Regarding treatment options, it is essential to educate both the patient and family about the causes and management of PNES. Shen et al. propose guidelines for discussing a diagnosis of PNES with the patient, including the recognition of PNES and the importance of close psychiatric follow-up [16]. An additional consideration for this patient is the importance of adhering to antipsychotic medication to prevent psychiatric decompensation and the return of distressing auditory hallucinations, which could trigger another PNES episode. Given the patient’s history of type I diabetes mellitus and a prior insulin-aided suicide attempt, the family was also educated on recognizing the differences in presentation between a PNES episode and a hypoglycemic state. There are many modalities for psychological intervention in treating patients with PNES. Cognitive behavioral therapy was recommended for this patient as it is efficacious in treating PNES [17]. In addition, physicians should recommend psychotherapy as well as education on adopting healthy coping mechanisms, such as mindfulness meditation, to deal with ongoing stressors [16,18].

## Conclusions

Patients with PNES often require a lengthy diagnostic workup to rule out similar presenting conditions such as epileptic seizure, hypoglycemic coma, and catatonia. Patients with comorbidities such as major depression and schizophrenia require a more nuanced approach to treatment. This often includes a combination of antipsychotic medication and adopting healthy coping mechanisms to prevent PNES recurrence. Finally, in patients with type I diabetes mellitus and a prior insulin-aided suicide attempt, it is essential to involve the patient and their family in recognizing signs and symptoms of PNES to distinguish these symptoms from a hypoglycemic seizure or coma.
